# Community-acquired pneumonia in children — a changing spectrum of disease

**DOI:** 10.1007/s00247-017-3827-8

**Published:** 2017-09-21

**Authors:** David M. le Roux, Heather J. Zar

**Affiliations:** 1 5th Floor ICH Building Red Cross War Memorial Children’s Hospital, Klipfontein Road Cape Town, 7700, South Africa; 2 Department of Paediatrics, New Somerset Hospital, Cape Town, South Africa

**Keywords:** Children, Conjugate vaccination, Epidemiology, *Haemophilus influenzae*, Incidence, Pneumococcus, Pneumonia, Radiology, Vaccination

## Abstract

Pneumonia remains the leading cause of death in children outside the neonatal period, despite advances in prevention and management. Over the last 20 years, there has been a substantial decrease in the incidence of childhood pneumonia and pneumonia-associated mortality. New conjugate vaccines against *Haemophilus influenzae* type b and *Streptococcus pneumoniae* have contributed to decreases in radiologic, clinical and complicated pneumonia cases and have reduced hospitalization and mortality. The importance of co-infections with multiple pathogens and the predominance of viral-associated disease are emerging. Better access to effective preventative and management strategies is needed in low- and middle-income countries, while new strategies are needed to address the residual burden of disease once these have been implemented.

## Introduction

Pneumonia has been the leading cause of death in children younger than 5 years for decades. Although there have been substantial decreases in overall child mortality and in pneumonia-specific mortality, pneumonia remains the major single cause of death in children outside the neonatal period, causing approximately 900,000 of the estimated 6.3 million child deaths in 2013 [1]. Substantial advances have occurred in the understanding of risk factors and etiology of pneumonia, in development of standardized case definitions, and in prevention with the production of improved vaccines and in treatment. Such advances have led to changes in the epidemiology, etiology and mortality from childhood pneumonia. However in many areas access to these interventions remains sub-optimal, with large inequities between and within countries and regions. In this paper we review the impact of recent preventative and management advances in pneumonia epidemiology, etiology, radiologic presentation and outcome in children.

## Epidemiology and burden of pneumonia in childhood

The overall burden of childhood pneumonia has been reduced substantially over the last decade, despite an increase in the global childhood population from 605 million in 2000 to 664 million in 2015 [2]. Recent data suggest that there has been a 25% decrease in the incidence of pneumonia, from 0.29 episodes per child year in low- and middle-income countries in 2000, to 0.22 episodes per child year in 2010 [3]. This is substantiated by a 58% decrease in pneumonia-associated disability-adjusted life years between 1990 and 2013, from 186 million to 78 million as estimated in the Global Burden of Disease study [1]. Pneumonia deaths decreased from 1.8 million in 2000 to 900,000 in 2013 [1]. These data do not reflect the full impact of increasingly widespread use of pneumococcal conjugate vaccine in low- and middle-income countries because the incidence of pneumonia and number of deaths are likely to decrease still further as a result of this widespread intervention [4].

Notwithstanding this progress, there remains a disproportionate burden of disease in low- and middle-income countries, where more than 90% of pneumonia cases and deaths occur. The incidence in high-income countries is estimated at 0.015 episodes per child year, compared to 0.22 episodes per child year in low- and middle-income countries [3]. On average, 1 in 66 children in high-income countries is affected by pneumonia per year, compared to 1 in 5 children in low- and middle-income countries. Even within low- and middle-income countries there are regional inequities and challenges with access to health care services: up to 81% of severe pneumonia deaths occur outside a hospital [5]. In addition to a higher incidence of pneumonia, the case fatality rate is estimated to be almost 10-fold higher in low- and middle-income countries as compared to high-income countries [3, 5].

Childhood pneumonia can also lead to significant morbidity and chronic disease. Early life pneumonia can impair long-term lung health by decreasing lung function [6]. Severe or recurrent pneumonia can have a worse effect on lung function; increasing evidence suggests that chronic obstructive pulmonary disease might be related to early childhood pneumonia [7, 8]. A meta-analysis of the risk of long-term outcomes after childhood pneumonia categorized chronic respiratory sequelae into major (restrictive lung disease, obstructive lung disease, bronchiectasis) and minor (chronic bronchitis, asthma, abnormal pulmonary function) groups [9]. The risk of developing at least one of the major sequelae was estimated as 6% after an ambulatory pneumonia event and 14% after an episode of hospitalized pneumonia. Because respiratory diseases affect almost 1 billion people globally and are a major cause of mortality and morbidity [10], childhood pneumonia might contribute to substantial morbidity across the life course.

## Changes in presentation of radiologic pneumonia

Chest radiologic changes have been considered the gold standard for defining a pneumonia event [11] because clinical findings can be subjective and clinical definitions of pneumonia can be nonspecific. In 2005, to aid in defining outcomes of pneumococcal vaccine studies, the World Health Organization’s (WHO) standardized chest radiograph description defined a group of children who were considered most likely to have pneumococcal pneumonia [12]. The term “end-point consolidation” was described as a dense or fluffy opacity that occupies a portion or whole of a lobe, or the entire lung. “Other infiltrate” included linear and patchy densities, peribronchial thickening, minor patchy infiltrates that are not of sufficient magnitude to constitute primary end-point consolidation, and small areas of atelectasis that in children can be difficult to distinguish from consolidation. “Primary end-point pneumonia” included either end-point consolidation or a pleural effusion associated with a pulmonary parenchymal infiltrate (including “other” infiltrate).

Widespread use of pneumococcal conjugate vaccination and *Haemophilus influenzae* type B conjugate vaccination has decreased the incidence of radiologic pneumonia. In a review of four randomized controlled trials and two case–control studies of *Haemophilus influenzae* type B conjugate vaccination in high-burden communities, the vaccination was associated with an 18% decrease in radiologic pneumonia [13]. Introduction of pneumococcal conjugate vaccination was associated with a 26% decrease in radiologic pneumonia in California between 1995 and 1998 [14]. In vaccine efficacy trials in low- and middle-income countries, pneumococcal conjugate vaccination reduced radiologic pneumonia by 37% in the Gambia [15], 25% in South Africa [16] and 26% in the Philippines [17].

The WHO radiologic case definition was not intended to distinguish bacterial from viral etiology but rather to define a sub-set of pneumonia cases in which pneumococcal infection was considered more likely and to provide a set of standardized definitions through which researchers could achieve broad agreement in reporting chest radiographs. However, despite widespread field utilization, there are concerns regarding inter-observer repeatability. There has been good consensus for the description of lobar consolidation but significant disagreement on the description of patchy and perihilar infiltrates [18, 19]. In addition, many children with clinically severe lung disease do not have primary end-point pneumonia: in one pre-pneumococcal conjugate vaccination study, only 34% of children hospitalized with pneumonia had primary end-point pneumonia [20]. A revised case definition of “presumed bacterial pneumonia” has been introduced, and this definition includes pneumonia cases with WHO-defined alveolar consolidation, as well as those with other abnormal chest radiograph infiltrates and a serum C-reactive protein of at least 40 mg/L [21, 22]. This definition has been shown to have greater sensitivity than the original WHO radiologic definition of primary end-point pneumonia for detecting the burden of pneumonia prevented by pneumococcal conjugate vaccination [23]. Using the revised definition, the 10-valent pneumococcal conjugate vaccine (pneumococcal conjugate vaccination-10), had a vaccine efficacy of 22% in preventing presumed bacterial pneumonia in young children in South America [22], and pneumococcal conjugate vaccination-13 had a vaccine efficacy of 39% in preventing presumed bacterial pneumonia in children older than 16 weeks who were not infected with human immunodeficiency virus (HIV) in South Africa [21]. Thus there is convincing evidence that pneumococcal conjugate vaccination decreases the incidence of radiologic pneumonia; however there is no evidence to suggest that pneumococcal conjugate vaccination modifies the radiologic appearance of pneumococcal pneumonia.

Empyema is a rare complication of pneumonia. An increased incidence of empyema in children was noted in some high-income countries following pneumococcal conjugate vaccination-7 introduction, and this was attributed to pneumococcal serotypes not included in pneumococcal conjugate vaccination-7, especially 3 and 19A [24]. In the United States, evidence from a national hospital database suggests that the incidence of empyema increased 1.9-fold between 1996 and 2008 [25]. In Australia, the incidence rate ratio increased by 1.4 times when comparing the pre-pneumococcal conjugate vaccination-7 period (1998 to 2004) to the post-pneumococcal conjugate vaccination-7 period (2005 to 2010) [26]. In Scotland, incidence of empyema in children rose from 6.5 per million between 1981 and 1998, to 66 per million in 2005 [27]. These trends have been reversed since the introduction of pneumococcal conjugate vaccination-13. Data from the United States suggest that empyema decreased by 50% in children younger than 5 years [28]; similarly, data from the United Kingdom and Scotland showed substantial reduction in pediatric empyema following pneumococcal conjugate vaccination-13 introduction [29, 30].

Several national guidelines from high-income countries, as well as the WHO recommendations for low- and middle-income countries, recommend that chest radiography should not be routinely performed in children with ambulatory pneumonia [31–33]. Indications for chest radiography include hospitalization, severe hypoxemia or respiratory distress, failed initial antibiotic therapy, or suspicion for other diseases (tuberculosis, inhaled foreign body) or complications. However, point-of-care lung ultrasound is emerging as a promising modality for diagnosing childhood pneumonia [34].

## Changes in hospitalization and incidence

In addition to the effect on radiologic pneumonia, pneumococcal conjugate vaccination reduces the risk of hospitalization from viral-associated pneumonia, probably by reducing bacterial–viral co-infections resulting in severe disease and hospitalization [35]. An analysis of ecological and observational studies of pneumonia incidence in different age groups soon after introduction of pneumococcal conjugate vaccination-7 in Canada, Italy, Australia, Poland and the United States showed decreases in all-cause pneumonia hospitalizations ranging from 15% to 65% [36]. In the United States after pneumococcal conjugate vaccination-13 replaced pneumococcal conjugate vaccination-7, there was a further 17% decrease in hospitalizations for pneumonia among children eligible for the vaccination, and a further 12% decrease among unvaccinated adults [28].

## Changing etiology of childhood pneumonia

A systematic review of etiology studies prior to availability of new conjugate vaccines confirmed *S. pneumoniae* and *H. influenzae* type B as the most important bacterial causes of pneumonia, with *Staphylococcus aureus* and *Klebsiella pneumoniae* associated with some severe cases. Respiratory syncytial virus was the leading viral cause, identified in 15–40% of pneumonia cases, followed by influenza A and B, parainfluenza, human metapneumovirus and adenovirus [37].

More recent meta-analyses of etiology data suggest a changing pathogen profile, with increasing recognition that clinical pneumonia is caused by the sequential or concurrent interaction of more than one organism. Severe disease in particular is often caused by multiple pathogens. With high coverage of pneumococcal conjugate vaccination and *Haemophilus influenzae* type B conjugate vaccination, viral pathogens increasingly predominate [38]. In recent case–control studies, at least one virus was detected in 87% of clinical pneumonia cases in South Africa [39], while viruses were detected in 81% of radiologic pneumonia cases in Sweden [40]. In a large multi-center study in the United States, viral pathogens were detected in 73% of children hospitalized with radiologic pneumonia, while bacteria were detected in only 15% of cases [41]. A meta-analysis of 23 case–control studies of viral etiology in radiologically confirmed pneumonia in children, completed up to 2014, reported good evidence of causal attribution for respiratory syncytial virus, influenza, metapneumovirus and parainfluenza virus [42]. However there was no consistent evidence that many other commonly described viruses, including rhinovirus, adenovirus, bocavirus and coronavirus, were more commonly isolated from cases than from controls. Further attribution of bacterial etiology is difficult because it is often not possible to distinguish colonizing from pathogenic bacteria when they are isolated from nasal specimens [43].

Another etiology is pertussis. In the last decade there has also been a resurgence in pertussis cases, especially in high-income countries [44]. Because pertussis immunity after acellular pertussis vaccination is less long-lasting than immunity after wild-type infection or whole-cell vaccination, many women of child-bearing age have waning pertussis antibody levels. Their infants might therefore be born with low transplacental anti-pertussis immunoglobulin G levels, making them susceptible to pertussis infection before completion of the primary vaccination series [45]. In 2014, more than 40,000 pertussis cases were reported to the Centers for Disease Control and Prevention in the United States; in some states, population-based incidence rates are higher than at any time in the last 70 years [44]. In contrast, most low- and middle-income countries use whole-cell pertussis vaccines and the numbers of pertussis cases in those countries were stable or decreasing until 2015 [46]. However recent evidence from South Africa (where the acellular vaccine is used) shows an appreciable incidence of pertussis among infants presenting with acute pneumonia: 2% of clinical pneumonia cases among infants enrolled in a birth cohort were caused by pertussis [39], and 3.7% of infants and young children presenting to a tertiary academic hospital had evidence of pertussis infection [47].

Similarly, childhood tuberculosis is a major cause of morbidity and mortality in many low- and middle-income countries, and *Mycobacterium tuberculosis* has increasingly been recognized as a pathogen in acute pneumonia in children living in high tuberculosis-prevalence settings. Postmortem studies of children dying from acute respiratory illness have commonly reported *M. tuberculosis* [48, 49]. A recent systematic review of tuberculosis as a comorbidity of childhood pneumonia reported culture-confirmed disease in about 8% of cases [50]. Because intrathoracic tuberculosis disease is only culture-confirmed in a minority of cases, the true burden could be even higher; tuberculosis could therefore be an important contributor to childhood pneumonia incidence and mortality in high-prevalence areas.

## Changing risk factors for childhood pneumonia

Childhood pneumonia and clinically severe disease result from a complex interaction of host and environmental risk factors [37]. Because of the effectiveness of pneumococcal conjugate vaccination and *Haemophilus influenzae* type B conjugate vaccination for prevention of radiologic and clinical pneumonia, incomplete or inadequate vaccination must be considered as a major preventable risk factor for childhood pneumonia. Other risk factors include low birth weight, which is associated with 3.2 times increased odds of severe pneumonia in low- and middle-income countries, and 1.8 times increased odds in high-income countries [51]. Similarly, lack of exclusive breastfeeding for the first 4 months of life increases odds of severe pneumonia by 2.7 times in low- and middle-income countries and 1.3 times in high-income countries. Markers of undernutrition are strong risk factors for pneumonia in low- and middle-income countries only, with highly significant odds ratios for underweight for age (4.5), stunting (2.6) and wasting (2.8). Household crowding has uniform risk, with odds ratios between 1.9 and 2.3 in both low- and middle-income countries and high-income countries. Indoor air pollution from use of solid or biomass fuels increases odds of pneumonia by 1.6 times; lack of measles vaccination by the end of the first year of age increases odds of pneumonia by 1.8 times [51]. It is estimated that the prevalence of these critical risk factors in low- and middle-income countries decreased by 25% between 2000 and 2010, contributing to reductions in pneumonia incidence and mortality in low- and middle-income countries, even in countries where conjugate vaccines have not been available [3].

The single strongest risk factor for pneumonia is HIV infection, which is especially prevalent in children in sub-Saharan Africa. HIV-infected children have 6 times increased odds of developing severe pneumonia or of death compared to HIV-uninfected children [52]. Since the effective prevention of mother-to-child transmission of HIV, there is a growing population of HIV-exposed children who are uninfected; their excess risk of pneumonia, compared to HIV unexposed children, has been described as 1.3- to 3.4-fold higher [53–57].

## Prevention strategies: new advances

The pneumococcal conjugate vaccination and *Haemophilus influenzae* type B conjugate vaccination have been effective tools to decrease pneumonia incidence, severity and mortality [58, 59]. However, equitable coverage and access to vaccines remains sub-optimal. By the end of 2015, *Haemophilus influenzae* type B conjugate vaccination had been introduced in 73 countries, with global coverage estimated at 68%. However, inequities are still apparent among regions: in the Americas coverage is estimated at 90%, while in the Western Pacific it is only 25%. By 2015, pneumococcal conjugate vaccination had been introduced into 54 countries, with global coverage of 35% for three doses of pneumococcal conjugate vaccination for infant populations [60]. To address this issue, the WHO’s Global Vaccine Access Plan initiative was launched to make life-saving vaccines more equitably available. In addition to securing guarantees for financing of vaccines, the program objectives include building political will in low- and middle-income countries to commit to immunization as a priority, social marketing to individuals and communities, strengthening health systems and promoting relevant local research and development innovations [61].

Maternal vaccination to prevent disease in the youngest infants has been shown to be effective for tetanus, influenza and pertussis [62]. Influenza vaccination during pregnancy is safe, provides reasonable maternal protection against influenza, and also protects infants for a limited period from confirmed influenza infection (vaccine efficacy 63% in Bangladesh [63] and 50.4% in South Africa [64]). However as antibody levels drop sharply after birth, infant protection does not persist much beyond 8 weeks [65]. Recently respiratory syncytial virus vaccination in pregnancy has been shown to be safe and immunogenic, and a phase-3 clinical trial of efficacy at preventing respiratory syncytial virus disease in infants is under way [66]. Within a decade, respiratory syncytial virus in infancy might be vaccine-preventable, with further decreases in pneumonia incidence, morbidity and mortality [67].

Improved access to health care, better nutrition and improved living conditions might contribute to further decreases in childhood pneumonia burden. The WHO Integrated Global Action Plan for diarrhea and pneumonia highlights many opportunities to protect, prevent and treat children [68]. Breastfeeding rates can be improved by programs that combine education and counseling interventions in homes, communities and health facilities, and by promotion of baby-friendly hospitals [69]. Improved home ventilation, cleaner cooking fuels and reduction in exposure to cigarette smoke are essential interventions to reduce the incidence and severity of pneumonia [70, 71]. Prevention of pediatric HIV is possible by providing interventions to prevent mother-to-child transmission [72]. Early infant HIV testing and early initiation of antiretroviral therapy and cotrimoxazole prophylaxis can substantially reduce the incidence of community-acquired pneumonia among HIV-infected children [73]. Community-based interventions reduce pneumonia mortality and have the indirect effect of improved-care-seeking behavior [58]. If these cost-effective interventions were scaled up, it is estimated that 67% of pneumonia deaths in low- and middle-income countries could be prevented by 2025 [58].

## Management — recent advances

Case management of pneumonia is a strategy by which severity of disease is classified as severe or non-severe. All children receive early, appropriate oral antibiotics, and severe cases are referred for parenteral antibiotics. When implemented in high-burden areas before the availability of conjugate vaccines, case management as part of Integrated Management of Childhood Illness was associated with a 27% decrease in overall child mortality, and 42% decrease in pneumonia-specific mortality [74]. However the predominance of viral causes of pneumonia and low case fatality have prompted concern about overuse of antibiotics. Several randomized controlled trials comparing oral antibiotics to placebo for non-severe pneumonia have been performed [75–77] and others are ongoing [78]. In two studies, performed in Denmark and in India, outcomes of antibiotic and placebo treatments were equivalent [76, 77]. In the third study, in Pakistan, there was a non-significant 24% vs. 20% rate of failure in the placebo group, which was deemed to be non-equivalent to the antibiotic group [75]. Furthermore, because WHO-classified non-severe pneumonia and bronchiolitis might be considered within a spectrum of lower respiratory disease, many children with clinical pneumonia could actually have viral bronchiolitis, for which antibiotics are not beneficial [79]. This has been reflected in British [33] and Spanish [31] national pneumonia guidelines, which do not recommend routine antibiotic treatment for children younger than 2 years with evidence of pneumococcal conjugate vaccination who present with non-severe pneumonia. The United States’ national guidelines recommend withholding antibiotics in children up to age 5 years presenting with non-severe pneumonia [32]. However, given the high mortality from pneumonia in low- and middle-income countries, the lack of easy access to care, and the high prevalence of risk factors for severe disease, revised World Health Organization pneumonia guidelines still recommend antibiotic treatment for all children who meet the WHO pneumonia case definitions [80].

Use of supplemental oxygen is life-saving, but this is not universally available in low- and middle-income countries; it is estimated that use of supplemental oxygen systems could reduce mortality of children with hypoxic pneumonia by 20% [81]. Identifying systems capacity to increase availability of oxygen in health facilities, and identifying barriers to further implementation are among the top 15 priorities for future childhood pneumonia research [82]. However, up to 81% of pneumonia deaths in 2010 occurred outside health facilities [5], so there are major challenges with access to health services and health-seeking behavior of vulnerable populations. Identifying and changing the barriers to accessing health care is an important area with the potential to impact the survival and health of the most vulnerable children [82].

## Conclusion

Much progress has been made in decreasing deaths caused by childhood pneumonia. Improved socioeconomic status and vaccinations, primarily the conjugate vaccines (against *Haemophilus influenzae* and pneumococcus), have led to substantial reductions in the incidence and severity of childhood pneumonia. Stronger strategies to prevent and manage HIV have reduced HIV-associated pneumonia deaths. However, despite the substantial changes in incidence, etiology and radiology globally, there remain inequities in access to care and availability of effective interventions, especially in low- and middle-income countries. Effective interventions need to be more widely available and new interventions developed for the residual burden of childhood pneumonia.
